# Safety of propofol-assisted deep extubation in the dental treatment of children—a retrospective, observational study

**DOI:** 10.1186/s12871-024-02599-2

**Published:** 2024-06-29

**Authors:** Xiang Zhang, Xiao-Dong Wang, Wei Cui, Shun-Cai Gao, Xu-Dong Yang, Bin Xia

**Affiliations:** 1 Department of Anesthesiology, Peking University School and Hospital of Stomatology, Beijing, 100081 China; 2 Department of Pediatric Dentistry, The No. 2 Hospital of Baoding, Baoding, 071051 China; 3 Department of Anesthesiology, Aerospace Center Hospital, Beijing, 100049 China; 4 Department Head of Anesthesiology, Peking University School and Hospital of Stomatology, Beijing, 100081 China; 5 Department Head of Pediatric Dentistry, Peking University School and Hospital of Stomatology, Beijing, 100081 China

**Keywords:** Intratracheal extubation, Mouth rehabilitation, General anesthesia

## Abstract

**Purpose:**

Awake extubation and deep extubation are commonly used anesthesia techniques. In this study, the safety of propofol-assisted deep extubation in the dental treatment of children was assessed.

**Materials and methods:**

Children with severe caries who received dental treatment under general anesthesia and deep extubation between January 2017 and June 2023 were included in this study. Data were collected on the following variables: details and time of anesthesia, perioperative vital signs, and incidence of postoperative complications. The incidence of laryngeal spasm (LS) was considered to be the primary observation indicator.

**Results:**

The perioperative data obtained from 195 children undergoing dental treatment was reviewed. The median age was 4.2 years (range: 2.3 to 9.6 years), and the average duration of anesthesia was 2.56 h (range 1 to 4.5 h). During intubation with a videoscope, purulent mucus was found in the pharyngeal cavity of seven children (3.6%); LS occurred in five of them (2.6%), and one child developed a fever (T = 37.8 °C) after discharge. Five children (2.6%) experienced emergence agitation (EA) in the recovery room. Also, 13 children (6.7%) experienced epistaxis; 10 had a mild experience and three had a moderate experience. No cases of airway obstruction (AO) and hypoxemia were recorded. The time to open eyes (TOE) was 16.3 ± 7.2 min. The incidence rate of complications was 23/195 (11.8%). Emergency tracheal reintubation was not required. Patients with mild upper respiratory tract infections showed a significantly higher incidence of complications (*P* < 0.001).

**Conclusions:**

Propofol-assisted deep extubation is a suitable technique that can be used for pediatric patients who exhibited non-cooperation in the outpatient setting. Epistaxis represents the most frequently encountered complication. Preoperative upper respiratory tract infection significantly increases the risk of complications. The occurrence of EA was notably lower than reported in other studies.

## Introduction

The optimal time for extubation is when the patient is either deeply anesthetized or fully awake [1, 2]. However, both strategies have certain risks. The complications of awake extubation include hypertension, arrhythmia, recurrent cough, laryngeal spasm, nausea, and vomiting, etc. These issues can be avoided or reduced through deep extubation performed by proficient anesthesiologists [3]. The complications of deep extubation include glossoptosis and pulmonary aspiration, among others, which can be avoided through awake extubation. Some researchers recommend performing deep extubation in pediatric patients aiming to lower overall airway issues, with the exception of airway obstruction [1–3].

Uncooperative children undergoing dental treatment in the outpatient pediatric department often express high sensitivity and anxiety. In such cases, ambulatory anesthesia is required, involving premedication for sedation and general anesthesia (GA) [4]. This procedure may coincide with a high rate of emergence agitation (EA), a complication that occurs during early recovery from sevoflurane anesthesia with an incidence rate of up to 80% [5]. Deep extubation can significantly alleviate EA [6].

In most studies, deep extubation cases require the use of sevoflurane [7–10]. Deep extubation assisted by propofol is infrequently reported. In a retrospective observational study, we tested the hypothesis that deep extubation assisted by the administration of intravenous propofol would be a suitable option for outpatient dental procedures in children who may not cooperate. Moreover, we have pinpointed factors that play a role in post-extubation complications.

## Materials and methods

### Study design

This retrospective, observational study was approved by the Ethics Committee of Peking University Hospital of Stomatology (PKUSSIRB-202,496,009). As this was an observational study and interventions were absent, the Ethics Committee waived the requirement for informed consent.

The study comprised outpatient pediatric department cases involving uncooperative children who underwent dental treatments under general anesthesia from January 2017 to June 2023.

The inclusion criterion was as follows:

1. Uncooperative children (defined as the child is not able to cooperate with the pediatric dentistry to complete the scheduled treatment plan, and the specialist has assessed that his/her dental problems requires dental treatment and the uncooperative behavior will endanger the safety of the treatment, and is not suitable for protective stabilization) who were above two years old undergoing GA with deep extubation performed by the senior author.

The exclusion criterion was as follows:

1. Patients with awake extubation, including those with American Society of Anesthesiologists(ASA) grade III or higher, airway conditions like severe adenoid or tonsillar hypertrophy, sleep apnea, micrognathia, difficult airway issues, and morbid obesity defined as a BMI z-score above 3 standard deviations from the median for those under 5 years and above 2 standard deviations for those over 5 years [11]; and recent upper respiratory tract infection.
2. Unknown method of extubation.

### Anesthesia procedure

Induction was achieved using 8–5% end-tidal sevoflurane (ET(sevo)), facilitating venepuncture. Tracheal intubation was conducted after administering 2–4 mg/kg of propofol intravenously and applying lidocaine jelly over the tracheal tube cuff. Subsequently, dexamethasone (0.1 mg/kg IV) and flurbiprofen lipid (1 mg/kg IV) were administered.

For maintenance, ETsevo (1–2%) and propofol (2–4 mg/kg*h) were used. Spontaneous respiration was ensured throughout the surgery, maintaining an end-tidal carbon dioxide (ETco_2_) between 45 and 55 mmHg, tidal volume (VT) of 6–8 mL/kg, and a respiratory rate (RR) of 14–18 breaths per minute. The dental procedures performed included extraction, root canal therapy, and cavity filling. A rubber dam was utilized to isolate most external water and debris. Local anesthesia was given before any painful stimuli.

At almost the end of treatment, oxygen was passed at a high flow rate (8–10 L/min) to replace sevoflurane. A saliva ejector was used to clear pharyngeal secretions and hematocele while the patient was in a lateral decubitus position. Propofol was continuously administered (8–10 mg/kg*h) after extubation, which was performed only when ETsevo readings were between 0.1% and 0.2% and no coughing occurred after the patients were positioned in the lateral decubitus position. In the recovery room, they received oxygen in the recovery room while remaining in the same position. SpO_2_ was continuously monitored until they were fully awake. The patients’ parents were present at the bedside throughout their time in the recovery room. All patients were followed up for 24 h after discharge.

If laryngeal spasms occurred, 100% O_2_ was administered under positive pressure through a face mask and 1 mg/kg propofol was intravenously administered. Mild epistaxis did not pose a threat. In the event of severe epistaxis, an adrenaline-soaked cotton swab was applied, and the patient was positioned in the lateral decubitus position.

The anesthesia and dental treatment protocols outlined earlier are standardized at our hospital. The extubation methods, however, are selected individually by each anesthesiologist based on their expertise and judgment.

The data were collected on the following parameters: laryngeal spasm (LS) was the primary outcome; time to open eyes (TOE), oxygen saturation (SpO_2_) at specified intervals, incidence of EA, epistaxis, and airway obstruction (AO) were the secondary outcomes.

### Definitions of outcomes

Major postoperative complications included LS, EA, AO, and epistaxis. TOE was defined as the duration from extubation to the eye opening. EA was identified and measured using PAED scale (Table 1) [11, 12]. Epistaxis was documented following the criteria established by Sugiyama et al.(Table 2) [13]. Oxygen desaturation was described as an SpO_2_ level of 90–95%, whereas hypoxemia was defined as an SpO_2_ of 90% sustained for at least 10 s.

**Table 1:** The PAED scale

1. The child makes eye contact with the caregiver.
2. The child’s actions are purposeful.
3. The child is aware of his/her surroundings.
4. The child is restless.
5. The child is inconsolable.

Items 1, 2, and 3 are reversed scored as follows: 4 not at all, 3 just a little,2 quite a bit, 1 very much, 0 extremely. Items 4 and 5 are scored as follows: 0 not at all, 1 just a little, 2 quite a bit, 3 very much, 4 extremely. The scores of each item were summed to obtain a total PAED scale score from 0 to 20. The degree of emergence delirium increased directly with the total score. EA defined as a PAED score ≥ 10.

**Table 2:** Severity of Epistaxis during Intubation

Severity	Definition
None	No blood observed on either the surface of the tube or posterior pharyngeal wall
Mild	Blood apparent on the surface of the tube or posterior pharyngeal wall
Moderate	Pooling of blood on the posterior pharyngeal wall
Severe	Large amount of blood in the pharynx impeding nasotracheal intubation and necessitating urgent orotracheal intubation

### Statistical analysis

To mitigate the potentially confounding effects of various methods of deep extubation and address the uncertainty surrounding the assessment of deep anesthesia state, the analyses were limited to patients who received deep extubation from our senior author. An imputation program was not utilized for the data; discrepancies in sample size due to missing data have been noted.

All statistical analyses were performed using SPSS 27.0 (IBM, Armonk, NY, USA). The Kolmogorov-Smirnov test was used to assess the normality of the data distribution. Chi-square tests were applied to analyze differences in categorical variables, and Mann-Whitney U tests were used for continuous variables, comparing patients with and without complications. A P-value of less than 0.05 was considered statistically significant.

## Results

### Patients

**Fig. 1:**
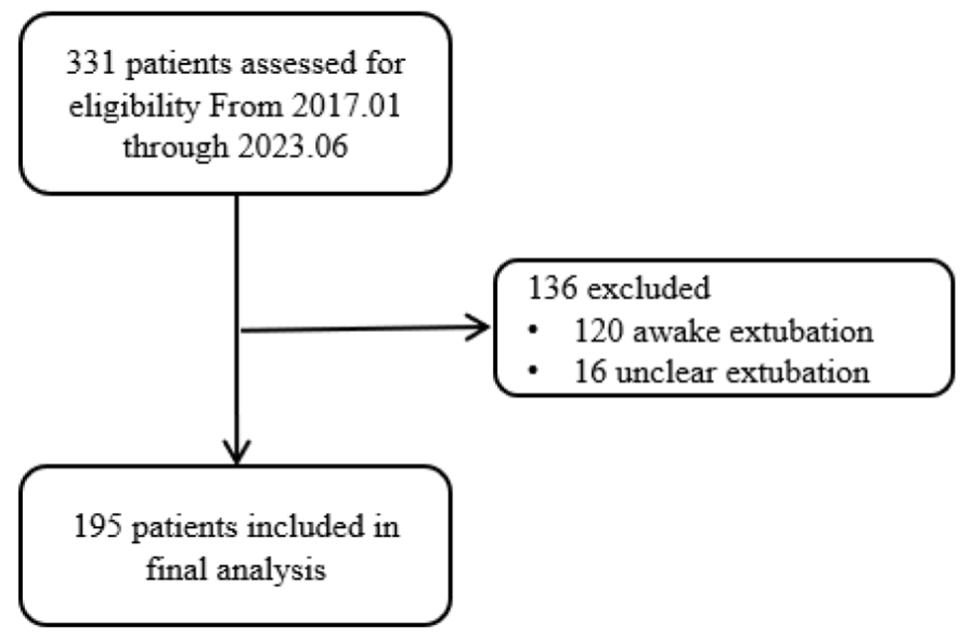
A flowchart of the study

We reviewed the perioperative data of 195 (out of 331) children undergoing deep extubation, which included 119 males and 76 females. A flowchart is presented in Fig. 1. The median age was 4.2 years (range: 2.3 years to 9.6 years). Among all cases, 168 cases (86.1%) were diagnosed as severe early childhood caries (SECC), including five cases (2.6%) with autism spectrum disorder.

### Outcome

The average duration of the procedure was 2.56 h (range 1 to 4.5 h). All patients underwent successful extubation, and emergency tracheal reintubation was not required. The TOE was 16.3 ± 7.2 min. Five children experienced LS after extubation, which was alleviated with 1.0 mg/kg propofol. Also, 13 children exhibited epistaxis, with 10 mild and 3 moderate cases. Adrenaline-soaked cotton swabs were used for hemostasis. Five children displayed EA (Table 3). The PAED scale sum score was 13.2 ± 1.17; the principal therapeutic intervention included parental consoling, while ice cream was the main therapy for treating sore throat. No instances of airway obstruction (AO) and hypoxemia were recorded. All children were safely discharged approximately 2 h after the operation.

**Table 3:** Complications after extuabtion

Complications	Yes(*n*)	No (*n*)
Emergence agitation	5	190
Laryngeal spasm	5	190
Glossoptosis	0	195
Epistaxis	13(10 mild,3 moderate)	182

The Mann-Whitney U test outcomes showed no significant variances in age, treatment duration, and the number of treated teeth between patients who experienced complications and those who did not. (Table 4). According to the Chi-square test results, mild upper respiratory tract infection was identified as the primary cause (χ^2^ = 19.229, *P* < 0.001) (Table 5). During intubation with a videoscope, purulent mucus was found in the pharyngeal cavity of all five children, and one of them developed a fever (T = 37.8 °C) after discharge.

**Table 4:** The results of the univariate analysis of complications

Complication	Age(years)	Anesthesia duration	Number of treated teeth
Yes	4.2(3.8;5.5)	2.50(2.10;3.42)	13.2(10.9;16.6)
No	4.1(3.9;6.0)	2.62(1.2;4.21)	14.3(8.6;17.9)
P	0.850	0.238	0.446

Values are presented as median (interquartile ranges at the 25th and 75th percentiles).

**Table 5:** The results of the univariate analysis of complications

Complication	Purulent Mucus	
	Yes	No
Yes	5	18
No	2	170
P	<0.001	

## Discussion

General anesthesia (GA) is preferred to deep intravenous propofol sedation for some reasons. The primary cause is the long duration of operation for full mouth rehabilitation, which also increases the chances of noncooperation and complications [4]. Additionally, GA is an alternative last resort strategy administered to autistic patients and those with intellectual disability. Protective stabilization and conscious sedation are not suitable for people with special needs (PSN) [14]. In the context of GA, we assessed the safety of propofol-assisted deep extubation in this study. Jeremy et al. [15]. examined factors influencing the outcomes of deep extubation in adults and noted that 40 out of 300 patients (13%) experienced at least one complication. In our study, the incidence of major postoperative complications was 11.8%, which was within the range reported by Kim et al. In a study, the desflurane-only group experienced a complication rate of 48%, whereas the desflurane combined with remifentanil group had a much lower complication rate of 3.4% [8]. This study also found that mild upper respiratory tract infection as the primary etioligy. Purulent mucus was observed in the pharyngeal cavity of all five children that developed into LS, whose white blood cell was within normal range and there was no fever, persistent cough, or running nose before surgery. Before intubation, vacuum aspiration of purulent mucus with saliva ejector can enhance the pharyngeal view, reduce the risk of airway complications, such as desaturation, laryngospasm and bronchospasm, etc.

A novel approach to propofol usage for deep extubation, which is infrequently documented, was implemented in this context [1, 8, 16, 17]. Although sevoflurane allows better intraoperative control of the depth of anesthesia and can prevent unintended regain of awareness, it is associated with EA (incidence rate of up to 80%) [18]. EA is a common complication that occurs after general anesthesia in children and has an incidence of 10–80% in children, depending on the different scoring systems, anesthetic techniques, and types of surgery [18–21]. In our study, we observed an incidence of emergence agitation (EA) at 2.6%, with propofol identified as the contributing factor [22–26]. In a study on pediatric patients, propofol was found to have a preventive effect against EA based on the timing of its administration [26]. Administering a 2 mg/kg intravenous bolus of propofol immediately after anesthesia induction did not reduce EA following desflurane anesthesia. Conversely, either continually infusing propofol during anesthesia maintenance or providing a propofol bolus at the conclusion of surgery exhibited a preventive influence on EA in pediatric patients under general anesthesia, aligning with our anesthesia protocol. The quick pharmacokinetics of propofol mainly account for these observed effects. Propofol has antiemetic effects at the level of GA. It has a shorter recovery time without persistent residual anesthetic effects. Additionally, it is cost-effective and easy to administer and titrate using steady-state infusion devices [4, 17]. More evidence is needed to contrast incidence of EA between different anesthetic techniques or sedatives.

Laryngeal spasm (LS) can manifest during extubation procedures, whether conducted awake or under deep anesthesia, irrespective of whether a laryngeal mask airway (LMA) or tracheal intubation is utilized. In our study, LS occurred in 2.6% of cases, a figure comparable to the reported incidence of respiratory critical events (3.1%, 95% CI: 2.9–3.3) from recent European data in the APRICOT study [27]. Researchers often consider LS as extended glottic closure due to intense stimulation either at the glottic or supraglottic regions. LS typically manifests as a sustained tonic adductor spike activity [28] and an increase in the tension of the submental muscle group. In clinical terms, complete laryngospasm is identified by chest wall movement without audible breathing, no visible bag movement, and ineffective ventilation. Partial laryngospasm is recognized by chest wall movement accompanied by stridulous breathing, where there is a partial synchronization between the patient’s respiratory efforts and the minimal bag movement [29]. We reduced the incidence of laryngospasm in our anesthesia procedure using various techniques, including applying lidocaine jelly over the tracheal tube cuff, IV dexamethasone application, performing oral suctioning before extubation, administering propofol continuously until complete elimination of sevoflurane, and performing deep extubation in lateral decubitus position. The conventional treatment of LS involves applying CPAP with 100% O_2_ and airway maneuvers. If initial interventions fail to resolve laryngospasm, succinylcholine (1–2 mg/kg) is administered, possibly with atropine (0.02 mg/kg in case of bradycardia), to alleviate the condition [29, 30]. The effect of intravenous (i.v.) anesthesia on LS needs to be further investigated. Our results showed that propofol can break laryngospasm, as shown in other studies [4, 30–32], suggesting that it is a feasible alternative.

Pediatric patients require higher oxygen, have lower functional residual capacity, and show higher CO_2_ ratios than adults. These differences make them more susceptible to perioperative hypoxia than adults [33]. In a study [34], Ungern-Sternberg found that tracheal extubation in fully conscious children was associated with a higher likelihood of continuous coughing. In contrast, children who were deeply anesthetized during extubation experienced a higher rate of airway obstructions, which could be managed by simple airway adjustments. Both extubation methods can be used to treat children at high risk undergoing adenotonsillectomy, however, rigorous postoperative monitoring is required. Our study suggests that the lateral decubitus position should be crucial for deep extubation [2, 35], which not only avoids glossoptosis but also prevents secretions or blood from entering the pharynx, thus, reducing the risk of aspiration or perioperative hypoxia. Placing the patient in a lateral position results in the expansion of the upper lung, leading to improved compliance in that area. As a result, a larger portion of the tidal volume is directed towards the upper lung, both during spontaneous breathing and mechanical ventilation. Larsson et al. [36]. 

discovered that the distribution of ventilation in anesthetized children placed in the lateral position mirrored previous findings in anesthetized adults, who also exhibited some degree of impaired pulmonary gas mixing. The functional residual capacity (FRC) notably increased when children were transitioned to the lateral position. Additionally, the lateral decubitus position provides a more comfortable sleeping position which means less oxygen consumption.

Although epistaxis cannot be completely avoided and may be life-threatening under deep extubation. In our study, incidence of epistaxis was 6.7%, which is much lower than R. Earle’s research(73% in the PFT group compared with 70% in the standard nasal RAE ETT group) [37]. Epistaxis was effectively managed using an adrenaline-soaked cotton swab and by putting the patient in the lateral decubitus position to avoid airway obstruction and pulmonary aspiration. Selecting the broader side of the nasal passage, using a slender tracheal tube according to Khine formulae (size(mm internal diameter) = 3 + Age(y)/4) [38–40], and implementing “gentle intubation techniques” may serve as better strategies to prevent epistaxis. Both intubation and extubation can lead to epistaxis. Further studies with larger groups of patients are needed to confirm whether deep extubation is related to less incidence of epistaxis.

Emergence hazards include hemodynamic changes, bleeding due to cough-induced agitation, airway irritation, significant body movement that can sometimes cause children to fall accidentally, etc. [41]. These issues can be minimized or reduced through deep extubation which is only performed by proficient anesthesiologists. The key points for deep extubation are described in Table 6.

**Table 6:** Key points for deep extubation

Key points For Deep Extubation
• Exclusion: micrognathia; severe adenoid or tonsillar hypertrophy; sleep apnea; morbid obesity. • Vacuum aspiration of upper respiratory tract secretions with saliva ejector • Intraoperation: Use of rubber dam, Continuous negative suction of secretion.
• Extubate in lateral decubitus position with the help of propofol when ETsevo reaches 0.1 ∼ 0.2%, BIS is below 70, and ETco_2_ is at 45–55 mmHg. Make sure no cough happening after placing the patients in lateral decubitus position, or IV propofol 1 mg/kg. • Accurately diagnose laryngeal spasm in advance (tension of the submental muscle group).
• Post operation: oxygen inhalation, lateral decubitus position, monitoring SpO_2_, parents at bedside.

Although deep extubation aims to decrease stress response during the recovery phase, the drawbacks of deep extubation cannot be overlooked, which include glossoptosis, apnea, emesis, laryngeal spasm, bronchospasm, pulmonary aspiration, etc. [42]. Goyagi et al. [43]. found that the incidence of respiratory complications such as apnea, laryngospasm, bronchospasm, and arrhythmias was not significantly different between deeply sevoflurane-anesthetized and awake children. Hence, special measures should be taken during deep extubation (Table 6). Patients presenting with severe adenoid or tonsillar hypertrophy, micrognathia, morbid obesity, sleep apnea, or any other indicators of difficult airways should be excluded from this technique. Utilization of a rubber dam is essential to isolate the treatment area, preventing the buildup of exogenous water and debris in the oropharynx, thereby reducing the risk of pulmonary aspiration. Placing the patient in the lateral decubitus position can aid in preventing glossoptosis and further reducing the risk of pulmonary aspiration. An oral airway should be placed in all adult patients [15]. Selecting the timing of extubation is also important. If a stable respiratory pattern can be maintained during deflation and reinflation of the endotracheal tube, using the cuff pressure is a widely accepted method to assess the depth of anesthesia [15, 44]. Orienting patients in the lateral decubitus position before extubation was our standard operation procedure and also our method to determine whether a patient was in a deep anesthesia state.

Deep extubation, however, cannot serve as a universal remedy for all scenarios [15]. Potential contraindications for deep extubation include patients with micrognathia, obesity, adenoidal hypertrophy, and adenoid face. Such patients should undergo extubation only after they recover from cough and swallow reflexes. In contrast, as deep extubation has a lower complication rate, it is more suitable for children with congenital heart disease, epilepsy, autism, and borderline intellectual functioning [2, 41, 45, 46]. For obese patients, implementing deep extubation via THRIVE might reduce desaturation episodes and unfavorable hemodynamic events, offering an alternative strategy for managing these patients [6].

This study was limited by its retrospective design at a single center and lack of a control group. Moving forward, randomized controlled trials are needed to explore the complications associated with deep extubation and assess the effects of different propofol dosages. Additionally, while deep extubation is considered suitable for autistic children and those with intellectual disabilities due to their heightened sensitivity to stimuli, further research is necessary to validate this assertion.

## Conclusions

Propofol-assisted deep extubation is a suitable technique that can be used for pediatric patients who exhibited non-cooperation in the outpatient setting. Epistaxis represents the most frequently encountered complication. Preoperative upper respiratory tract infection significantly increases the risk of complications. The occurrence of EA was notably lower than reported in other studies.

## Data Availability

The data used to support the findings of this study are available from the corresponding author upon request.
